# Impact of Insurance Status on Diagnostic Stage in Hodgkin’s Lymphoma in the United States: Implications for Detection and Outcomes

**DOI:** 10.7759/cureus.11600

**Published:** 2020-11-20

**Authors:** Daniel Rapado, Sean Chowdhari, Chan Gu, Marcia Varella, Grettel Castro, Pura Rodriguez de la Vega, Juan Lozano

**Affiliations:** 1 Department of Translational Medicine, Herbert Wertheim College of Medicine Florida International University, Miami, USA

**Keywords:** hodgkin lymphona, national cancer database and seer analyses, lack of health insurance, private health insurance, stage of diagnosis, social determinants of health, medicaid, medicare

## Abstract

Introduction and objective

Hodgkin’s lymphoma (HL) is a form of cancer originating from white blood cells that presents upon diagnosis with well-characterized symptoms (palpable lymph nodes, fever, night sweats, weight loss). HL is currently one of the most treatable cancers, with a successful treatment rate of 75% worldwide. The objective of this study is to evaluate the association between insurance status and the stage of diagnosis of HL in the United States from the years 2007 to 2016.

Methods

A cross-sectional study using secondary data from the Surveillance, Epidemiology, and End Results (SEER) program database was used. Insurance status of each patient was defined as uninsured (not insured or self-pay), any Medicaid (includes Indian/public health service), insured (private insurance, managed care, Health Maintenance Organization (HMO), Preferred Provider Organization (PPO), or Medicare) and insured not specified. Staging was dictated via the SEER combined/American Joint Committee on Cancer (AJCC) cancer staging guidelines. We divided the stages into early-stage (localized) and late-stage (regional by direct extension, involving distant sites/nodes). We used univariate descriptive analysis to determine baseline characteristics, bivariate analysis to evaluate potential confounding, and binary logistic regression to compute unadjusted and adjusted odd ratios and corresponding 95% confidence intervals.

Results

Approximately 77% of insured individuals presented with a late-stage diagnosis, compared with 78.1% for insured not specified, 82% for any Medicaid, and 84.9% for uninsured. After adjusting for age, sex, race and marital status, insurance status had a significant impact on the stage of diagnosis of Hodgkin's lymphoma. The odds ratio (OR) for advanced stage diagnosis of HL in uninsured patients compared to insured patients was 1.72 (95% CI 1.03-2.86, p=0.037); for any Medicaid, the OR was 1.37 (95% CI 1.02-1.83, p=0.036), and for insured not specified, 1.09 (95% CI 0.83-1.44, p=0.522).

Conclusions

Uninsured patients are significantly more likely to have a later stage diagnosis of HL compared to those that are insured. The findings of this study coincide with the associations found in previous studies involving other cancers such as breast, cervical, prostate, colorectal, hepatocellular, bladder and kidney cancers outcomes and insurance status.

## Introduction

Hodgkin’s lymphoma (HL) is a form of cancer originating from white blood cells that presents upon diagnosis with well-characterized symptoms (palpable lymph nodes, fever, night sweats, weight loss). Lymphoma is the most common form of cancer in adolescents from the ages of 15-19. HL makes up two-thirds of these cases, accounting for 0.5% of all newly diagnosed cases of cancer among all individuals in the US. Additionally, HL is currently one of the most treatable cancers, with a successful treatment rate of 75 percent worldwide. Successful treatment is defined as complete remission, usually following six cycles of either adriamycin, bleomycin, vinblastine, and dacarbazine (ABVD) or mechlorethamine, vincristine, procarbazine and prednisone (MOPP) regimens [1]. However, its associated risk factors are much less well-explained, and there currently does not exist any effective screening test or early detection method. 

Prior studies have characterized the influence of various socioeconomic and racial disparities on HL survival rates. With respect to insurance status, patients who are uninsured or have public insurance present with worse HL-specific survival rates compared to those with private or military insurance [2]. This coincides with the notion that adults who lack health insurance are more likely to delay or skip medical care due to financial issues and, as a result, are at increased risk for poorer health outcomes [3]. 

Additionally, earlier stages of HL at diagnosis are associated with better survival outcomes. According to the Surveillance, Epidemiology, and End Results (SEER) database between 1998 and 2014, the five-year survival for patients with localized HL was 15% greater than for those with distant staged [4]. A major concern for patients with late-stage HL is the increased risk of developing aggressive large diffuse B cell lymphoma [1]. The stage of diagnosis is also important as it dictates treatment and prognosis. Patients with an early stage will usually only receive abbreviated courses of combined modality chemotherapy/radiation therapy, while those with an advanced stage will receive more prolonged combination chemotherapy with radiation only in specific cases [5]. Additionally, uninsured patients have reduced access to screening and preventative services which may affect health outcomes [6]. These screening services, such as mammography, colonoscopies and Pap smears, are essential for early detection and prevention. While there is no current screening test for HL, access to healthcare services can be beneficial in detecting early symptoms which lead to earlier stages at diagnosis. 

Several studies have found an association between lack of adequate health insurance and advanced stages of cancer at diagnosis. A study by Davis et al. showed that women in a tertiary cervical cancer center with private insurance were significantly more likely to be diagnosed with cervical cancer at an earlier stage (9.1%) and age (41 y/o) compared to uninsured women (37.1% and 49 y/o, respectively, p<0.01) [7]. Another study by Halpern et al. discovered that in breast cancer patients, uninsured and Medicaid patients had an OR of 1.5 (p<.001) for stage II breast cancer at diagnosis and an OR of 2.4 (p<.001) for stages III/IV versus stage I compared to non-Medicaid insured patients [8]. Two studies compared insurance status to some of the most common and deadly cancers in the U.S., such as breast, lung, and prostate. Both studies found a higher OR for uninsured and non-Medicaid insured patients for late-stage cancer diagnosis [9-10]. However, there is currently a lack of research regarding the association between health insurance status and stage of HL at the time of diagnosis, which is what this study aims to address. Findings of this study can be applicable towards identifying modalities to improve outcomes in terms of the diagnosis and prognosis of HL.

HL has several possible risk factors, including prior Epstein-Barr virus (EBV) infection, age, gender, and family history; however, the associations between these risk factors and the likelihood of a later diagnosis are not clearly characterized [11]. In addition, there are currently no widely recommended screening tests for HL outside of attentiveness towards common early signs and symptoms, which include palpable lymph nodes, fever, night sweats, and weight loss. This study will attempt to distinguish possible disparities regarding stage at diagnosis that may be consequent to differences in insurance coverage between individuals. Conclusions of the study can be applicable towards improving overall detection and outcomes in HL patients at risk for late diagnosis through efforts including but not limited to improved awareness and early intervention.

## Materials and methods

Study design

A cross-sectional study using secondary data collected between 2007 and 2016. 

Database

We utilized secondary data from the Surveillance, Epidemiology, and End Results (SEER) program database. The SEER database collects and publishes cancer incidence and survival data yearly from various cancer registries over 13 states which cover a third of the U.S. population. SEER registry databases contain information regarding patient demographics, primary tumor site, tumor morphology and stage at diagnosis, first course of treatment, and follow-up for vital status. Cancer mortality data in the SEER database is obtained from the National Center for Health Statistics. Population data for cancer incidence and demographic characteristics are obtained from the Census Bureau. 

Population and sample

Our sample consisted of individuals who were diagnosed with HL from 2007-2016 in the United States identified in the SEER database. Individuals were chosen from 2007 and onwards as the SEER database only began tracking insurance status starting in 2007. Additionally, no restrictions on the age of our population were implemented as HL is bimodal and affects both adolescents and the elderly [1]. 

Patients were included in this study if they had been diagnosed with any stage of HL after 2007 in the U.S in the following areas: thymus, mediastinum, spleen, bone marrow, hematopoietic, lymph nodes of head face and neck, lymph nodes of arm, thoracic lymph nodes, intra-abdominal lymph nodes, multiple region lymph nodes and tonsils. Patients were excluded from this study if either insurance status or stage of Hodgkin’s disease were unknown. 

Variables 

The independent variable of the study was insurance status, which was classified into the categories of uninsured (not insured or self-pay), any Medicaid (includes Indian/public health service), insured (private insurance such as fee-for-service, managed care, Health Maintenance Organization (HMO), Preferred Provider Organization (PPO), or Medicare), and insured not specified, all based on the SEER Insurance Recode. 

HL staging was the outcome measure, as dictated by the SEER combined/American Joint Committee on Cancer (AJCC) cancer staging guidelines. Staging was categorized into early (localized) or late (regional by direct extension, involving distant sites/nodes). Localized is defined by SEER as a single lymph node involved or multiple nodal chains in the same region. For extranodal lymphomas, it involves only a single extralymphatic site without nodal involvement or multifocal involvement of an extralymphatic organ without nodal involvement. Regional by direct extension is defined as bulky disease present in two or more lymph node region on the same side of the diaphragm or contiguous extension between extralymphatic sites. Distant sides/nodes is defined as diffuse involvement of one or more extralymphatic sites, or involvement of lymph node regions on both sides of the diaphragm. Variables considered for potential confounding include age, sex, race (White, Black, and other), and marital status (married and not married, which included single, separated, divorced, widowed, unmarried or domestic partner). 

Statistical analysis 

An initial univariate descriptive analysis on the baseline characteristics of our population consisting of age, sex, insurance status and stage of diagnosis was conducted. For quantitative variables we used measurements means and standard deviations. Categorical qualitative variables were described using percentages. A bivariate analysis was then conducted to determine the association between baseline characteristics with both insurance status and HL stage at diagnosis. Variables showing a significant association with both insurance status and HL stage at diagnosis were considered potential confounders. A collinearity analysis was used to assess independence of all independent variables. Lastly, binary logistic regression was conducted to determine the association between insurance status and HL staging at diagnosis while controlling for potential confounders, obtaining unadjusted and adjusted odds ratios and 95% confidence intervals. 

## Results

**Figure 1:**
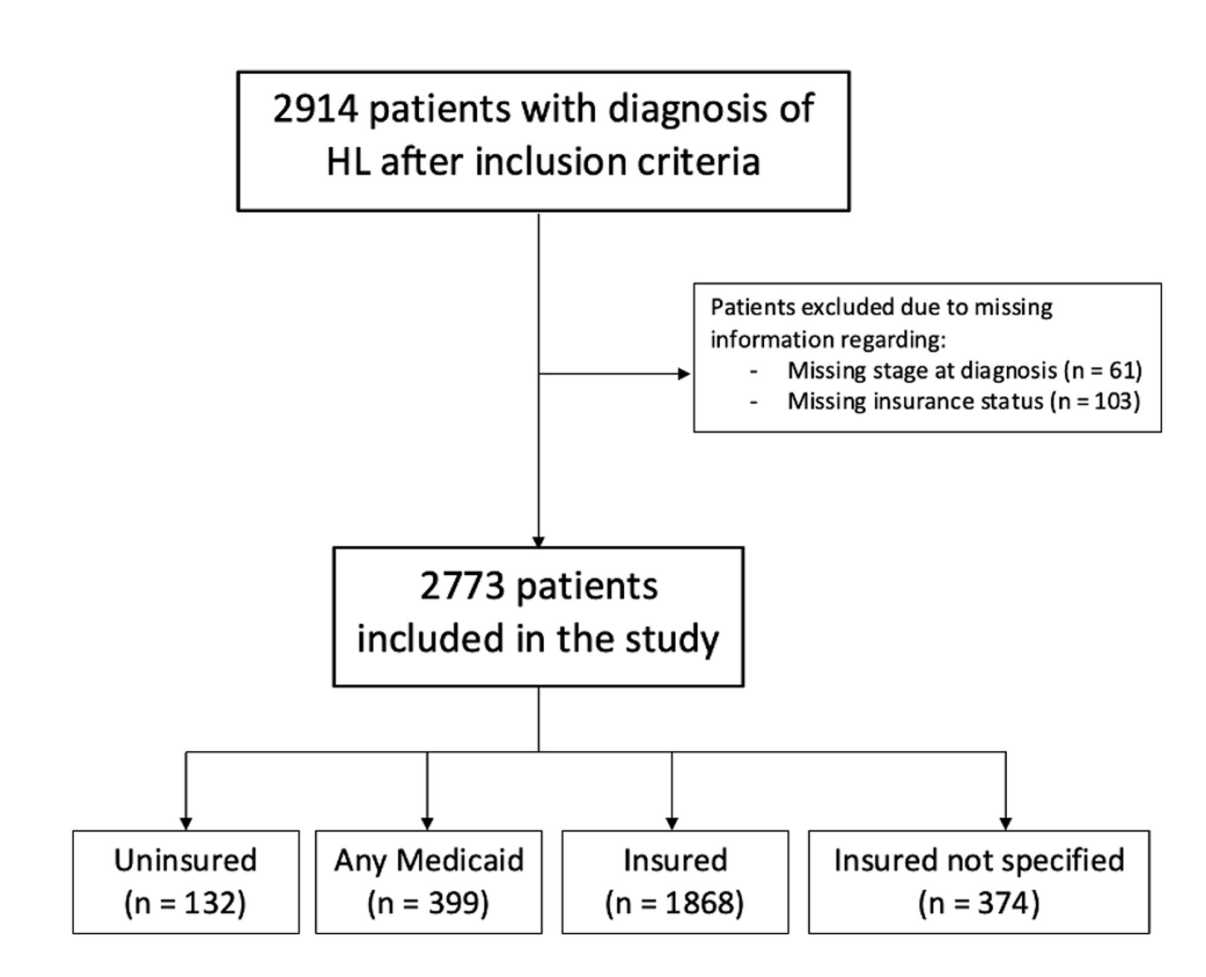
Flow chart of sample population selection with application of inclusion and exclusion criteria.

Application of the inclusion criteria yielded 2,914 patients. Sixty-one were excluded from the study due to missing the stage at diagnosis of their HL, and 103 patients for missing insurance status at time of diagnosis. There were some patients who both had missing stage of diagnosis and missing insurance status. After the exclusion criteria were applied, 2,773 patients remained in the study (Figure 1). 

Table 1 shows the baseline characteristics of our sample. It mostly consisted of white (75.3%) and male (62.4%) patients who were insured (80.9%). Patients in the uninsured and any Medicaid groups were younger than those with any form of insurance. Most of the individuals under the insured and insured non specified categories classified as married, while a large majority of individuals under the uninsured and any Medicaid categories classified as not married. The uninsured and the any Medicaid groups had higher frequencies of Black or Other race patients than the insured and the insured non specified groups.

**Table 1:** Characteristics of Patients with Hodgkin’s Lymphoma With Varying Degrees of Insurance *P < 0.05 indicates significance.

Characteristics	Uninsured		Any Medicaid		Insured		Insured non specified		P value
	N	%	N	%	N	%	N	%	
Age (y) - Mean (SD)	34.4 (13.4)		37.3 (18.1)		45.7 (20.4)		49.2 (20.4)		<0.001*
Sex									
Male	93	70.5	251	62.9	1145	61.3	241	64.4	0.149
Female	39	29.6	148	37.1	723	38.7	133	35.6	
Marital Status									
Married	26	21.1	75	19.5	932	52.5	184	51.8	<0.001*
Not Married	97	78.9	309	80.4	845	47.6	171	48.2	
Race									
White	77	58.8	256	65.3	1463	79	292	78.3	<0.001*
Black	51	38.9	109	27.8	254	13.7	61	16.4	
Other	3	2.3	27	6.9	135	7.3	20	5.4	

We found significant differences between the rate of early- vs. late-stage diagnosis among different insurance groups (Table 2). Approximately 77% of insured individuals presented with a late-stage diagnosis, compared with 78.1% for insured not specified, 82% for any Medicaid, and 84.9% for uninsured. With respect to gender, a statistically significant percentage of females presented with late-stage diagnoses (80.6%), compared to 76.8% for males. Although not statistically significant, unmarried individuals had a higher rate of presenting with late-stage diagnoses, 78.5%, compared to 77.4% to married individuals. Finally, regarding race, whites had the lowest percentage of late-stage diagnoses, at 77.6%, compared to 79.4% for blacks and 81.6% for others (not statistically significant). 

**Table 2:** Relationship between Patient Characteristics and Stage of Diagnosis *P < 0.05 indicates significance.

Characteristics	Early stage		Late stage		p-value
	N	%	N	%	
Insurance					
Uninsured	20	15.2	112	84.9	0.042*
Any Medicaid	72	18.1	327	82.0	
Insured	429	23.0	1439	77.0	
Insured not specified	82	21.9	292	78.1	
Age (y) - Mean (SD)	44.2 (20.0)		44.4 (20.2)		0.834
Sex					
Male	401	23.2	1329	76.8	0.018*
Female	202	19.4	841	80.6	
Marital Status					0.478
Married	275	22.6	942	77.4	
Not married	305	21.4	1117	78.5	
Race					
White	467	22.4	1621	77.6	0.36
Black	98	20.6	377	79.4	
Other	34	18.4	151	81.6	

Table 3 shows the unadjusted and adjusted odds ratios for the associations between our baseline characteristics and a late-stage diagnosis of Hodgkin’s lymphoma. These results illustrate insurance status having a significant impact on the stage of diagnosis of Hodgkin’s lymphoma. When compared to patients with insurance, those who lacked insurance and who had Medicaid had a significant increase in the odds of late-stage diagnosis (70% and 40%, respectively); in contrast, there was no significant difference in the frequency of late diagnosis between patients with insurance and who were insured not specified. Although no confounders were suspected after the bivariate analyses, we further adjusted for age, sex, race, and marital status, and continued to find that insurance status has a significant impact on the stage of diagnosis. Following this adjustment, we found that the odds ratio for advanced-stage diagnosis of HL in uninsured patients compared to insured patients remained the same as well as for any Medicaid. In the case of the insured not specified category, the odds increased to 10%.

**Table 3:** Unadjusted and Adjusted Odds Ratios for the Association between Baseline Characteristics and Late Stage of Diagnosis of Hodgkin’s Lymphoma *P < 0.05 indicates significance.

Characteristics	Unadjusted		Adjusted	
	OR (95% CI)	p-value	OR (95% CI)	p-value
Insurance				
Insured	Ref		Ref	
Uninsured	1.7 (1.02-2.7)	0.039	1.7 (1.03-2.9)	0.037*
Any Medicaid	1.4 (1.03-1.8)	0.032	1.4 (1.02-1.8)	0.036*
Insured non specified	1.06(0.8-1.4)	0.661	1.1 (0.8-1.4)	0.522
Age (continuous)	1.0 (1.0-1.0)	0.834	1.0 (1.0-1.0)	0.445
Sex				
Male	Ref		Ref	
Female	1.3 (1.04-1.5)	0.019	1.3 (1.05-1.6)	0.016*
Marital Status				
Married	Ref		Ref	
Not Married	1.1 (0.9-1.3)	0.478	1.03 (0.8-1.3)	0.8
Race				
White	Ref		Ref	
Black	1.1 (0.9-1.4)	0.411	1.03 (0.8-1.3)	0.825
Other	1.3 (0.9-1.8)	0.211	1.3 (0.9-1.9)	0.196

## Discussion

Hodgkin’s lymphoma, though one of the most common types of cancers in adolescents, is also one of the most treatable types, with a worldwide successful treatment rate of over 75% [4]. Despite this, its risk factors remain poorly characterized, and there are currently no effective screening tests or early detection methods. 

The aim of this study was to evaluate the association between insurance status and the stage of diagnosis of Hodgkin’s lymphoma. We found that lack of insurance or being on Medicaid had a 70% and a 40% increase in the diagnosis of late-stage Hodgkin’s lymphoma when compared to insured patients, a finding that persisted following adjustment for characteristics including age, sex, race, and marital status. Our study contributes to the rapidly growing body of literature, which is currently predominated by studies depicting the influence of insurance status on survival rate and outcomes for many different types of cancers. 

The reasoning behind our proposed association between insurance coverage status and staging at diagnosis of Hodgkin’s lymphoma is as follows. The benefits of insurance coverage present as easier access to routine healthcare and medical care. With regards to different types of cancers, patients without these benefits are less likely to have access to cancer screening services and are more likely to present with an advanced stage disease at diagnosis [6]. It is more difficult for them to seek advice and routine care from a medical professional. In addition, lack of medical intervention leads to progression of disease, precipitating a higher stage at diagnosis. 

The causal mechanism that we propose to be responsible for this observation is that those who are uninsured tend to seek out health screenings at lower rates compared to those who have insurance due to financial or temporal constraints. Another possibility is that those who have purchased health insurance are more likely to be conscious of their health and will seek out medical help if they notice a change in their bodies such as with the symptoms of HL. With a treatable cancer such as HL, it is paramount to catch the early symptoms to increase the likelihood of survival. Our data also show that women were 30% more likely than men to present with a late-stage diagnosis of HL. This was a surprising association as it opposes the general stigma of male resistance to frequent and early medical care. 

Several studies have also characterized the association between health insurance coverage type and other forms of cancer and diseases. Zaydfudim et al. found that in hepatocellular carcinoma, patients without insurance were not only more likely to be diagnosed at later stages, but also less likely to receive surgical treatment and have double the disease-causing death rate (p<0.005) [12]. A study by Nguyen et al. found that as primary care provider density increases in an area, the overall amount of late-stage diagnosis of urologic cancers decreases. However, there remained a higher odds ratio of late-stage disease at diagnosis for uninsured patients compared to privately insured patients [13]. 

Our study used the SEER database, one of the most comprehensive cancer registries in the US, representing approximately 34.6% of the US population. Overall, the sample population covered by the SEER database is representative of the US population in terms of general demographics. In addition, several important variables, such as education status and poverty status are also comparable to those of the US general population. For instance, the percent of individuals below poverty level is 15.3% within SEER, compared to 15.1% in the US general population [14]. Additionally, the percent of individuals under 25 with less than a high school diploma is 14.2%, compared to 13.0% in the US general population. In addition, following the initial analysis, our study included an adjustment for several variables which included age, race, sex, and marital status. This helped control for confounders that may have been influenced by any of these variables.

Our study has several limitations, one of which is the use of the SEER database. Despite being one of the most comprehensive registries on cancer, containing data on approximately 34.6% of the US population, the data only comes from a select 13 of the 50 states. In addition, there are several demographic measurements that differ between the SEER dataset and the US census. The SEER dataset contains a slightly higher proportion of foreign-born individuals, 17.9%, compared to the US census measure of 13.2% [14]. The constraints of what variables the SEER database offers also limits our study in controlling for all possible confounding. Factors such as income and education level are not explicitly measured within the database but could have an impact on the association with insurance status and stage at diagnosis. 

Our study also suffers from a lack of uninsured individuals. Compared to the general US population, only 19% of our sample of patients was either uninsured or on Medicaid, a markedly lower proportion. In addition, it may be that Hodgkin’s lymphoma does present in all individuals at a similar stage, but uninsured individuals delay efforts to seek care despite exhibiting explicit symptoms. One reason for this may be an overall lack of awareness among certain populations. One explanation for this could be that insured individuals are more likely to have been exposed to information warning them about the early signs of Hodgkin’s lymphoma, and thus are more likely to immediately seek care once similar symptoms appear. This is open for further study as the mechanism is not clearly defined. Uninsured individuals, despite presenting with the classical symptoms, may not be aware of later consequences, and thus delay seeking care. Uninsured individuals are also less likely to have the time for more frequent physician visits, leading to further progression of the cancer before a diagnosis is made.

## Conclusions

Many previous studies have characterized the relationship between insurance status and cancer outcomes. In general, studies have shown that lack of insurance leads to increased mortality rates in the treatment of several types of cancers such as breast, prostate, colorectal, cervical, hepatocellular, kidney and bladder cancer. Our research expands on this by addressing not the outcomes, but the initial stage at diagnosis of one specific type of cancer, Hodgkin’s lymphoma.

Following the results of our research, one recommendation could be that emphasis can be placed on spreading awareness of the early signs of Hodgkin’s lymphoma. Given the erratic presentation of this type of cancer, the discrepancy between stage of diagnosis among individuals of different insurance groups could be explained by a general lack of awareness of the classical initial findings and symptoms.

Future studies can attempt to focus on the reason why uninsured individuals tend to present at late stages with a cancer that does not have any clear risk factors or screening tests. They may also attempt to explore whether increasing education and awareness among certain population groups can lead to overall improvements, with an increase in the proportion of individuals present with an early stage at diagnosis.
